# ADENOCARCINOMA OF TRANSPOSED COLON: FIRST CASE OF SYNCHRONOUS TUMOR

**DOI:** 10.1590/S0102-67202014000200018

**Published:** 2014

**Authors:** Rubens Antonio Aissar SALLUM, Gilton Marques FONSECA, Sergio SZACHNOWICZ, Francisco Carlos Bernal da Costa SEGURO, Ivan CECCONELLO

**Affiliations:** Departamento de Gastroenterologia, Disciplina de Cirurgia do Aparelho Digestivo, Faculdade de Medicina da Universidade de São Paulo (Department of Gastroenterology, Digestive Surgery Division, University of São Paulo School of Medicine), São Paulo, SP, Brazil.

## INTRODUCTION

The surgical and anatomical basis for using the colon as a substitute for the esophagus were established in 1911 by Kelling and Vuillet^1^ and for many years was the technique of choice for esophageal replacement^2^. Its use is helpful in benign diseases, such as caustic or peptic strictures, and malignancies^1,3^, especially when the stomach cannot be used, and also in children with congenital anomalies^2,4^. However, this procedure is subject to early complications, as ischemia of the colon and leakage^5^, or late problems as anastomosis stenosis, ischemic colitis, fistula due to diverticulitis and malignant lesions^4^.

The transposed colon cancer is a rare complication. Since 2007, six new cases were reported and two reviews published. Hwang et al^6^ found 10 reported cases of adenocarcinoma in the transposed colon and Bando et al^7^ also reviewed 10 cases in the literature, encompassing adenomas and adenocarcinomas.

The aim is to report an unique case of synchronous adenocarcinoma of the transposed colon.

## CASE REPORT

**Figure 1:**
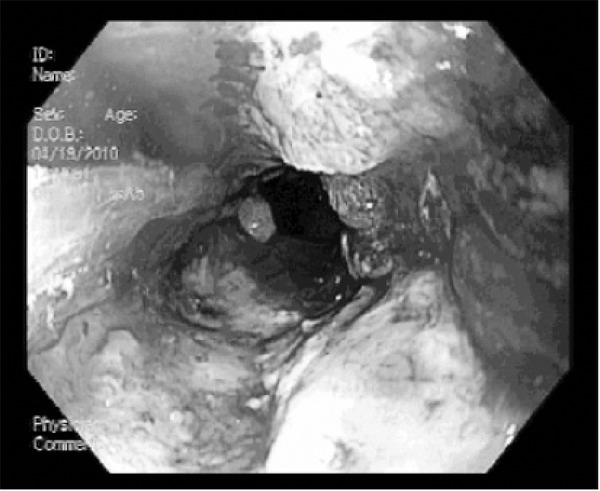
Endoscopic view of the stenotic area in proximal colonic segment with advanced adenocarcinoma

**Figure 2:**
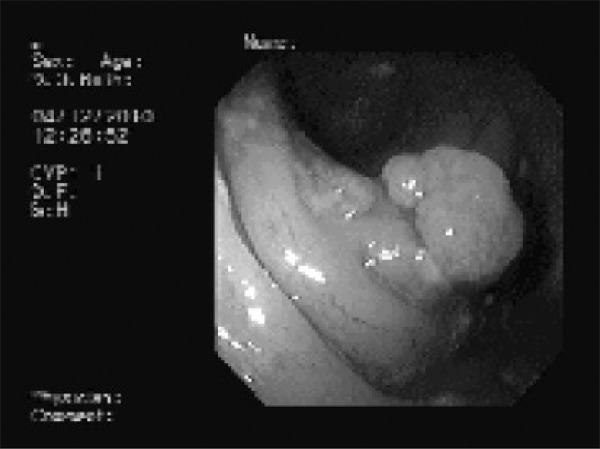
Endoscopic view of the sessile polyp with sincronous intramucosal adenocarcinoma at the transposed colon more distal

Woman with 53-years-old diagnosed with congenital esophageal atresia, underwent to several surgical procedures in childhood, the latest was a cervical retrosternal esophagocoloplasty at 11 years old. After 42 years she was evolved with cervical dysphagia, and an initial diagnosis of stenosis of the esophagocolic anastomosis was performed, treated with endoscopic dilation without improvement. Later, biopsies were performed in the area of ​​stenosis in proximal colonic segment (Figure 1) and polypectomy of sessile polyp of 10 mm, 5 cm distal to the stenosis (Figure 2). The pathological assessment showed tubular-villous intramucosal adenocarcinoma at the resected polyp and the area of ​​stenosis was a invasive adenocarcinoma in colonic mucosa. Colonoscopy of remained colon was normal. Staging performed with CT scan showed an eccentric wall thickening of proximal colon transposed with luminal reduction target of left innominate vein; densification of mediastinal fat plane adjacent and regional lymph nodes up to 1.9 cm.

Surgical treatment was performed with neck incision, sternotomy and laparotomy with resection of the colon transposed and a tactic transhiatal esophagectomy of the atresic esophagus in order to pull up the greater curvature gastric conduit obtained by the posterior mediastinum route. Resection of a portion of the left innominate vein which was invaded by the tumor was also performed. The pathological examination of surgical specimen showed moderately differentiated tubular adenocarcinoma invading pericolical tissues and the left innominate vein, with no affected lymph nodes - p T4 N0 (0 / 42) M0.

The patient developed postoperative superior vena cava syndrome, treated by anticoagulation. She had ischemia of the proximal portion of the stomach transposed being performed partial gastrectomy, and respiratory complications. She remained in intensive care and under multidisciplinary clinical support. Discharge of the hospital was after 128 days. Patient developed recurrent disease (lung metastases), started chemotherapy, and died nine months after surgery due to pneumonia.

## DISCUSSION

There are basically three options for replacement after esophageal resection: stomach, colon and small bowel^8^. For many years, the colon was considered the organ of choice, but the stomach has been the most widely used in recent decades due facility of preparation of the gastric conduit and its more robust vascular supply as a result of a rich submucosal vascular layer^9^. Resection of the gastric lesser curvature allows elongation and a safe cervical anastomosis^8,10,11^.

In cases of previous gastrectomy, gastric caustic or peptic strictures, tumor involvement of the stomach or failed gastroplasty the colon is used^9^. Colonic interposition may have early complications as transposed colon ischemia and anastomotic fistula. Late complications as anastomotic stricture "redundant graft", ulceration, colitis, perforation, diverticulitis, or tumor in the colonic segment are reported^4,5^. Must be remembered that colorectal cancer has a high incidence; is the third leading cause of cancer diagnosed in men and second among women in the world^12^ and this colonic segment has a risk for malignancy too. There are 21 cases of adenoma/adenocarcinoma in transposed colon described in literature^1,3-7^.

This case shows that all patient underwent to esophagocoloplasty and develops dysphagia during late follow-up should be investigated for malignancy and the initial diagnosis of stenosis of the esophagocolic anastomosis without biopsy should be evoid.
